# Age-Related Changes in the Fraction of Cervical Intraepithelial Neoplasia Grade 3 Related to HPV Genotypes Included in the Nonavalent Vaccine

**DOI:** 10.1155/2019/7137891

**Published:** 2019-11-06

**Authors:** Luca Giannella, Giovanni Delli Carpini, Jacopo Di Giuseppe, Sonia Prandi, Dimitrios Tsiroglou, Andrea Ciavattini

**Affiliations:** ^1^Woman's Health Sciences Department, Gynecologic Section, Polytechnic University of Marche, Ancona, Italy; ^2^AUSL-IRCCS of Reggio Emilia, Cervical Cancer Screening Centre, Reggio Emilia, Italy

## Abstract

**Objective:**

The prevalence of some human papillomavirus (HPV) genotypes has been shown to change with age. So, also the distribution of HPV genotypes included in the nonavalent vaccine may not be the same at all ages, and this could mean that vaccine protection against cervical cancer may be affected by age. The present study aimed to evaluate whether there are age-related changes in the fraction of high-grade cervical intraepithelial neoplasia (CIN) attributable to HPV genotypes included in the nonavalent vaccine.

**Methods:**

Two hundred four consecutive women undergoing conization with a histological diagnosis of CIN3 were retrospectively analyzed. All included women had a preconization HPV genotyping (HPV Sign® Genotyping Test). The women were divided into three groups according to age: <35, 35–44, and ≥45 years of age. Based on HPV genotypes detected in cervical lesions, the age-related changes in the expected vaccine protection were evaluated by the Cochran–Armitage test for trend.

**Results:**

The fraction of CIN3 attributable to HPV genotypes included in the nonavalent vaccine showed a significant negative trend with increasing age, with potential vaccine protection of 82% after the age of 45 (*p*=0.006). The rate of HPV-16 and HPV-33, included in the vaccine, showed a negative trend with age (*p*=0.047 and *p*=0.044, respectively). Among HPV genotypes not covered by the vaccine, the rate of non-high-risk HPVs (genotypes: 53-54-70-73-82-85-87) showed a significant positive trend with increasing age (*p*=0.018).

**Conclusions:**

Although the fraction of CIN3 attributable to genotypes included in the nonavalent HPV vaccine was high even after age 45, older women appeared to be more at risk of high-grade CIN related to HPV genotypes not included in the vaccine. Interestingly, older women showed a higher rate of precancerous cervical lesions associated with non-high-risk HPV. The present findings seem to raise the question about the management of cervical pathology at a later age in a future postvaccination era.

## 1. Introduction

Cervical cancer (CC) is one of the most common neoplasms among women worldwide, especially in developing countries [1]. Currently, anti-human papillomavirus (HPV) vaccination and cervical cancer screening programs represent the primary and secondary prevention strategies, respectively.

Concerning primary prevention, there are three types of HPV vaccines with a well-established safety profile [2]. The bivalent vaccine includes the high-risk (hr)-HPV genotypes 16 and 18 found in 70% of CCs; the quadrivalent vaccine comprises, in addition to the above-mentioned genotypes, the low-risk HPV-6 and -11 that cause 90% of genital warts [3, 4]; finally, the most recent nonavalent vaccine includes the hr-HPV genotypes 16, 18, 31, 33, 45, 52, and 58 and the low-risk HPV genotypes 6 and 11 [5]. In the latter case, with supposed prolonged immunogenicity, it was estimated that almost 90% of CCs could be prevented [6].

Although the vaccine protection is very high, it should be taken into account that the distribution of HPV genotypes is age-dependent [7]. Some HPV genotypes showed a negative or positive trend with age. Serrano et al. reported that the prevalence of HPV-16, -18, and -45 in CCs decreased with increasing age [1]; otherwise, the incidence of HPV genotypes 35, 52, 53, 56, 59, and 73 showed a positive trend with increasing age in women affected by CCs [1]. Finally, hr-HPV genotypes showed a negative trend with increasing age in women with cervical intraepithelial neoplasia (CIN) grade 3 [8]. Therefore, it would seem that the prevalence of HPV genotypes is not the same at all ages. In this regard, to assess whether expected HPV vaccine protection is affected by age may be a topic of interest for the management of cervical pathology.

Based on the question mentioned above, the present study aimed to evaluate whether there are age-related changes in the fraction of high-grade CIN related to HPV genotypes included in the nonavalent HPV vaccine.

## 2. Materials and Methods

### 2.1. Study Population and Methodology

The present retrospective observational study included women with CIN3 diagnosis on cone specimens at the Cervical Cancer Screening Centre of Reggio Emilia and University Hospital of Ancona, Italy, between September 2011 and November 2014. The present study was approved by the Local Ethical Committee (Comitato Etico Regionale Marche) with the following protocol number: N 2015 0486OR. All patients provided written informed consent for the use of their data for research purposes before any diagnostic or therapeutic procedure.

All included women were sent to colposcopy for an abnormal Pap smear and had a preconization diagnosis of CIN2+ on cervical biopsy or persistent low-grade CIN for at least two years. They had a presurgery HPV genotyping performed on the same day of conization, as a routine procedure, because our local protocols provided a different time interval between conization and the first subsequent colposcopic evaluation according to preconization HPV genotype. All included women had a histological diagnosis of CIN3 on cone specimen (the real precancer lesion). Women with an anti-HPV vaccination, previous conization, immunological disease, concomitant malignancies (including cervical cancer), or pregnancy were excluded. All data were retrieved from computerized software used in our Colposcopic Clinics.

Cervical conizations were performed in an outpatient setting with local anesthesia and under colposcopic guidance. Based on previous studies [9], HPV genotyping outcomes were classified as negative, positive for hr-HPV (genotypes 16, 18, 31, 33, 35, 39, 45, 51, 52, 56, 58, 59, 66, and 68), and positive for non-hr-HPV (probable high-risk HPV genotypes 26, 30, 53, 67, 70, 73, 82, and 85; and low-risk HPV genotypes 6, 11, 40, 42, 43, 44, 54, 55, 61, and 69). The rate of single and multiple HPV infections was measured. According to previous studies [10], when multiple HPV infections were present, a hierarchical attribution estimate was used. In this regard, CIN3 was attributed to the genotype most associated with high-grade cervical lesions or CCs. For example, a lesion was attributed to HPV genotypes not included in the vaccine only if HPV genotypes included in the vaccine were not present (HPV 16, 18, 31, 33, 45 52, and 58).

The women were divided into three categories according to age: <35 years of age, 35–44 years of age, and ≥45 years of age. The age-related changes in the fraction of CIN3 related to HPV genotypes included in the nonavalent HPV vaccine were evaluated by comparing the rate and trend between groups based on HPV genotypes detected in cervical lesions. Furthermore, the possible age-related changes of each HPV genotype included in the vaccine were evaluated (including its presence in both single and multiple infections). Finally, the rate and trend between age groups of HPV genotypes not included in the vaccine were assessed (hr-HPV genotypes and non-hr-HPV genotypes). All results from the three different age groups were expressed as numbers and percentages.

Given that HPV genotype distribution may be affected by age, ethnicity, and sample collection year, an univariate analysis was performed to assess the impact of these confounding variables.

### 2.2. Collected Samples

DNA was extracted from 204 human cervical samples and analyzed using the HPV Sign® Genotyping Test (Qiagen, Hilden, Germany). All samples were collected with an endocervical swab and Thin Prep (TP) (Hologic, Marlborough, MA, USA) the same day of conization before surgery. Twelve of these samples showed the absence of HPV DNA with a confirmed lesion on cone specimen. The latter samples underwent further molecular testing by cone biopsy.

### 2.3. DNA Isolation

For cytological samples, 4 mL of each specimen collected by TP was digested with 20 *μ*L of proteinase K in 180 *μ*L of lysis buffer (buffer ATL) at 56°C for one hour and then incubated in 200 *μ*L of lysis solution (buffer AL) at 70°C for 10 min. After that, 200 *μ*L of ethanol (96–100%) was added to each sample, followed by vortexing for 15 s. After briefly centrifuging the samples, they were washed repeatedly, and the DNA was eluted in 60 *μ*L of buffer and extracted with the QIAmp DNA Mini Kit (Qiagen, Hilden, Germany) following the manufacturer's instructions.

For tissue samples (cone specimens), DNA was extracted after deparaffinization as described for the cytologic samples. DNA was stored at −20°C until use.

### 2.4. HPV Sign® Genotyping Test

As previously described [11], this assay is based on the broad-spectrum amplification of HPV DNA using end-point PCR, with melting curve analysis performed on a Rotor-Gene Q Real-Time PCR Cycler (Qiagen, Hilden, Germany). Mixed primers targeting a hypervariable region of the HPV L1 ORF were used, and the *β*-globin gene was used as the internal control, followed by pyrosequencing with multiple sequencing primers. Melting curve analysis detected specific peaks for HPV sequences and the *β*-globin gene, allowing the semiquantitative determination of the presence or absence of HPV DNA. Only HPV-positive samples were further analyzed by pyrosequencing using the Pyromark Q24 System (Qiagen, Hilden, Germany) and four specific sequencing primers to identify the viral genotypes. These sequencing primers allowed the synthesis of genotype-specific sequences of 30 bases. Furthermore, they had a high discriminatory power for identifying the HPV genotype of each sample. IdentiFireTM software (version 1.0.5.0; Biotage AB, Uppsala, Sweden) was used to compare the obtained sequences with genotype-specific sequences in the HPV Sign® Q24 library. The assay was performed following the manufacturer's instructions.

### 2.5. Statistical Analysis

The Kolmogorov–Smirnov test was used to assess the distribution of continuous variables (age). Given that our data originated from ordered categories, to test the relationship between two classification factors (e.g., age and HPV genotypes), we used the chi-squared test for trend (or the Cochran–Armitage test for trend), which is more potent than the unordered independence test when a classification table has two columns and three or more rows (or two rows and three or more columns) [12].

All statistical analyses were performed using MedCalc Statistical Software version 19.0.3 (MedCalc Software bvba, Ostend, Belgium; https://www.medcalc.org; 2019). A *p* value <0.05 was considered statistically significant.

## 3. Results

**Figure 1:**
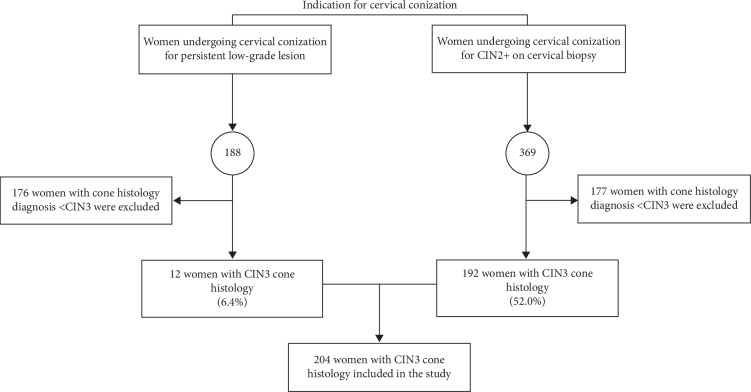
Study flowchart according to the indication for cervical conization.

Two hundred four consecutive women with a histological diagnosis of CIN3 on cone specimens were included in this retrospective study. A study flowchart, according to the indication for cervical conization, is shown in Figure 1.

The women were divided into three groups according to age: <35 years of age (79 women), 35–44 years of age (75 women), and ≥45 years of age (50 women). Preconization hr-HPV genotypes were detected in 196/204 (96.1%) women (with at least one hr-HPV type). Preconization non-hr-HPV genotypes were detected in 8/204 (3.9%) women. Single infections were found in 181 (88.7%) women. Multiple infections were found in 23 women (11.3%). Potential HPV vaccine protection was 91.7%. Patient characteristics are shown in Table 1.

**Figure 2:**
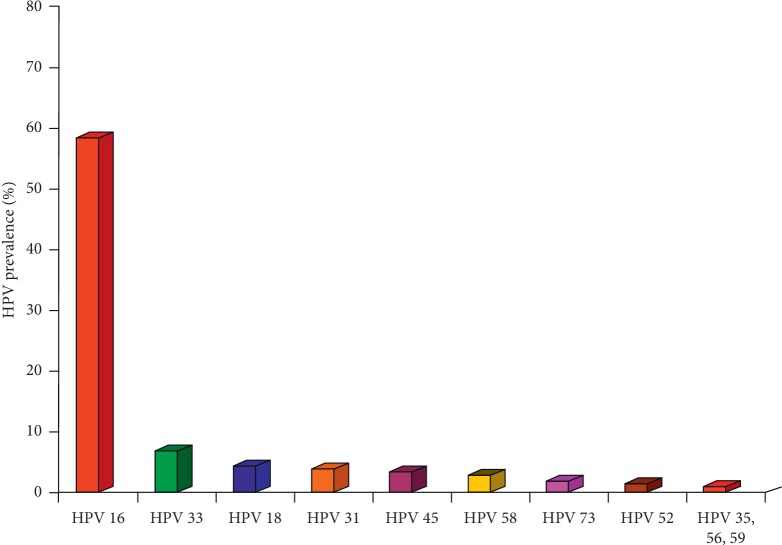
HPV genotype distribution in women with cervical intraepithelial neoplasia grade 3.

HPV genotype distribution in women with CIN3 is shown in Figure 2. The most common genotype was HPV-16 found in 119 women; HPV-33 was found in 14 women; HPV-18 was found in 9 women; HPV-31 was found in 8 women. The most common multiple infection was HPV-16 + HPV-18 found in 5 women.

The distribution of each HPV genotype included in the nonavalent vaccine was analyzed (Table 2). HPV-16 showed a significant negative trend with increasing age (*p*=0.047); HPV-33 showed a similar significant negative trend with increasing age (*p*=0.044). HPV-18, -31, -52, -45, and -58 showed no significant trend with age (Table 2). There was no woman with HPV-6 or HPV-11 infection, included in the vaccine.

The fraction of CIN3 attributable to HPV genotypes included in the nonavalent vaccine showed a significant negative trend with increasing age: 96.2% in women <35 years of age, 93.3% in women of 35–44 years of age, and 82.0% in women ≥45 years of age (*p*=0.006) (Table 3). There was no difference of HPV genotype distribution according to sample collection year and ethnicity (Table 3).

Women with HPV genotypes not covered by the nonavalent vaccine were 17 cases (8.3%). Hr-HPV genotypes: 3 women with HPV-59, 2 women with HPV-56, 2 women with HPV-35, 1 woman with HPV-39, and 1 woman with a multiple infection including HPV-66 + HPV-53; non-hr-HPV genotypes: 4 women with HPV-73, 1 woman with HPV-53, -87, and -82, and 1 woman with a multiple infection including HPV-70 + HPV-85. hr-HPV genotypes not included in the vaccine showed a positive but not significant trend with increasing age: 2.5% in women <35 years of age, 4.0% in women of 35–44 years of age, and 8.0% in women ≥45 years of age (*p*=0.153). The distribution of non-hr-HPV genotypes showed a significant positive trend with increasing age: 1.3% in women <35 years of age, 2.7% in women of 35–44 years of age, and 10.0% in women ≥45 years of age (*p*=0.018) (Table 3).

## 4. Discussion

The present study showed that the fraction of CIN3 lesions attributable to HPV genotypes included in the nonavalent vaccine decreased with increasing age. The same significant trend was observed for HPV genotypes 16 and 33, included in the vaccine. Conversely, among HPV genotypes not covered by the vaccine, non-hr-HPVs showed a significant positive trend with increasing age.

Nonavalent HPV vaccine was initially approved for women aged 9–26 years, and in October 2018, the Food and Drug Administration extended its use to men and women aged 27–45 years [13]. It has been estimated that this vaccine, if given before exposure to the included genotypes, can achieve cervical cancer protection close to 90% [14]. Overall, our results are in line with these data since the fraction of CIN3 attributable to the genotypes included in the vaccine was 91.7%. In a recent study evaluating the fraction of CIN2+ due to genotypes included in the nonavalent HPV vaccine, Perez et al. reported similar results with a percentage of CIN3-CIS covered by the vaccine of 86% [10].

The inclusion of specific HPV genotypes in the nonavalent vaccine has led to very high protection against precancerous and cancerous cervical lesions. However, several studies in the literature have shown that the prevalence of HPV genotypes, and therefore of those HPV-related cervical pathologies, varies with age [15, 16]. The distribution of HPV genotypes included in the nonavalent vaccine may not be the same at all ages, and this could mean that vaccine protection against high-grade CIN or cancer may be affected by age.

In a fascinating paper, Guardado–Estrada et al. showed that almost half of CCs in older women were due to non-hr-HPV [16]. Other studies showed that the prevalence of HPV-16 in high-grade CIN revealed a negative trend with age [15, 17]. As reported previously, it is likely that HPV-16 cervical lesions progress more rapidly, making their occurrence less frequent in older women [18]. Our results showed a similar negative trend with age of HPV-16 CIN3 lesions. Furthermore, the significant negative trend with age of HPV-16 and HPV-33 may explain the overall negative trend in expected vaccination protection reported by the present study. Before us, a previous study including 244 women with CIN3 also showed a significant negative trend with increasing age in the potential nonavalent HPV vaccine protection [10]. The authors demonstrated vaccine protection of 90/86/76% for CIN3/CIS in the same age groups (18–34, 35–44, and ≥45 years), respectively [10].

So, it would seem that older women are more at risk of high-grade cervical lesions due to genotypes not included in the nonavalent vaccine. These results may be a matter to be investigated for the future management of cervical cancer screening programs. There are studies in the literature that used simulation models to assess the appropriate screening intensity in vaccinated women. They showed that for women undergoing bivalent vaccine, including HPV-16 and 18 genotypes, three lifetime screens would be cost-effective; while for women subjected to the nonavalent vaccine, only two lifetime screens would be needed [19]. These results seem to suggest that screening intervals could be reviewed when vaccinated women will approach the age at which cervical screening begins. However, a possible revision of the cervical cancer screening recommendations based on the vaccination status should take into account these age-related changes in the prevalence of HPV genotypes. As reported by previous authors, the peak incidence of cervical cancer in the postvaccination era will be moved to a later age [20]. In this regard, older women could represent a population to be studied further for appropriate screening intervals and exit. Furthermore, it is well known that increasing age represents a limiting factor in the diagnostic assessment of CIN, and these further data should be kept in mind by colposcopists who manage cervical pathology [17, 21, 22].

A further result of the present study was that non-hr-HPV genotypes showed a significant positive trend with increasing age in CIN3 lesions. Although they represented a small percentage of cases, in this group of women, there was an increase of high-grade cervical lesions unrelated to hr-HPV. Interestingly, these women would have been negative at the primary HPV screening test that includes only hr-HPV genotypes. Currently, in Italy, only the HPV test is used in cervical screening program from 30 years of age. Based on these results, it would be interesting to ask the question about the need to use a cotesting (Pap smear + HPV test) at a given age cutoff. In a recent paper, Sun et al. showed that about 40% of older women with CIN2+ and carcinoma had a negative hr-HPV test, and these cases would have been missed without using the Pap test [23].

As reported previously, these age-related changes in HPV genotype distribution may be due to the woman's immunological status [24]. Older women undergo immune changes that can affect the acquisition or reactivation of HPV infections. It is likely that those less-common HPV genotypes, which are more easily cleared by a younger immune system, can result in persistent infections that progress to high-grade lesions in older women. Further studies investigating HPV genotype prevalence, age, and immunological factors should be performed to assess this hypothesis.

The present study has the limitation of being retrospective. Furthermore, although the group of women not covered by the vaccine provided significant results, it included a small sample of subjects. Not last, it must be taken into account that the hierarchical attribution of HPV genotypes may have led to an overestimation of vaccination protection. Conversely, it should be emphasized that we only included women with CIN3 that represent the real precancer cervical lesion. Furthermore, our histological reference standard was represented by cone specimens and not by cervical biopsies. Finally, HPV genotype sampling was carried out on the same day of conization, making the data very reliable.

## 5. Conclusions

To conclude, although the expected HPV vaccine protection against CIN3 was high even after age 45, the present results seem to raise the question about the management of cervical pathology at a later age in a future postvaccination era. Further studies with even larger sample sizes would be needed to confirm these results.

## Figures and Tables

**Table 1:** Patient characteristics.

Variables	Sample size (204) *n* (%)
Age (years) (median and interquartile range)	37.0 (31.0–44.0)
Ethnicity	
Italian	162 (79.4)
Chinese	11 (5.4)
East Europe	26 (12.7)
Northern Europe	1 (0.5)
African	4 (2.0)
Sample collection year	
2011 (4 months)	22 (10.8)
2012 (12 months)	64 (31.4)
2013 (12 months)	67 (32.8)
2014 (11 months)	51 (25.0)
Single HPV infections	181 (88.7)
Multiple HPV infections	23 (11.3)
Potential HPV vaccine protection	187 (91.7)
Non-high-risk HPV genotypes	8 (3.9)

HPV: human papillomavirus.

**Table 2:** Distribution of HPV genotypes included in the nonavalent HPV vaccine according to age groups in CIN3.

HPV genotypes	<35 years *n* (%)	35–44 years *n* (%)	≥45 years *n* (%)	*p* value^*∗*^
	Sample size (79)	Sample size (75)	Sample size (50)	
HPV-16	57 (72.2)	54 (72.0)	27 (54.0)	**0.047**
HPV-18	5 (6.3)	6 (8.0)	4 (8.0)	**0.697**
HPV-31	2 (2.5)	7 (9.3)	3 (6.0)	**0.303**
HPV-33	9 (11.4)	5 (6.7)	1 (2.0)	**0.044**
HPV-45	2 (2.5)	2 (2.7)	4 (8.0)	**0.148**
HPV-52	1 (1.3)	2 (2.7)	1 (2.0)	**0.713**
HPV-58	3 (3.8)	1 (1.3)	3 (6.0)	**0.624**

^*∗*^Using the Cochran–Armitage test for trend. HPV: human papillomavirus; CIN: cervical intraepithelial neoplasia.

**Table 3:** Distribution of HPV genotypes according to age, ethnicity, and sample collection year.

Variables	Nonavalent HPV vaccine genotypes *n* (%)	*p* value	Non-high-risk HPV genotypes *n* (%)	*p* value
Age (years)		**0.006**		**0.018**

<35 years	76 (96.2)		1 (1.3)	
35–44 years	70 (93.3)		2 (2.7)	
≥45 years	41 (82.0)		5 (10.0)	
Ethnicity		**0.902**		**0.726**

Italian	148 (91.4)		7 (4.3)	
Chinese	9 (81.8)		1 (9.1)	
East Europe	25 (96.2)		0 (0.0)	
Northern Europe	1 (100)		0 (0.0)	
African	4 (100)		0 (0.0)	
Sample collection year		**0.842**		**0.223**

2011 (4 months)	20 (90.9)		0 (0.0)	
2012 (12 months)	59 (92.2)		2 (3.1)	
2013 (12 months)	62 (92.5)		3 (4.5)	
2014 (11 months)	46 (90.2)		3 (5.9)	

HPV: human papillomavirus.

## Data Availability

The data used to support the findings of this study are available from the corresponding author upon request.
